# A Novel CD133- and EpCAM-Targeted Liposome With Redox-Responsive Properties Capable of Synergistically Eliminating Liver Cancer Stem Cells

**DOI:** 10.3389/fchem.2020.00649

**Published:** 2020-08-11

**Authors:** Zihua Wang, Mengqi Sun, Wang Li, Linyang Fan, Ying Zhou, Zhiyuan Hu

**Affiliations:** ^1^Key Laboratory of Brain Aging and Neurodegenerative Diseases of Fujian Provincial Universities and Colleges, School of Basic Medical Sciences, Fujian Medical University, Fuzhou, China; ^2^CAS Key Laboratory of Standardization and Measurement for Nanotechnology, CAS Key Laboratory for BiomedicalEffects of Nanomaterials and Nanosafety, CAS Center for Excellence in Nanoscience, National Center for Nanoscience and Technology of China, Beijing, China; ^3^Key Laboratory of Colloid Interface and Chemical Thermodynamics, Institute of Chemistry Chinese Academy of Sciences, Beijing, China; ^4^School of Nanoscience and Technology, Sino-Danish College, University of Chinese Academy of Sciences, Beijing, China

**Keywords:** targeting peptide, glutathione responsive, liver cancer stem cell, targeted drug delivery, synergistic therapy

## Abstract

Cancer stem cells (CSCs) are a small subset of cells that sit atop the hierarchical ladder in many cancer types. Liver CSCs have been associated with high chemoresistance and recurrence rates in hepatocellular carcinoma (HCC). However, as of yet, no satisfactorily effective liver CSC-targeted treatment is available, which drove us to design and investigate the efficacy of a liposome-based delivery system. Here, we introduce a redox-triggered dual-targeted liposome, CEP-LP@S/D, capable of co-delivering doxorubicin (Dox) and salinomycin (Sal) for the synergistic treatment of liver cancer. This system is based on the association of CD133- and EpCAM-targeted peptides to form Y-shaped CEP ligands that were anchored to the surface of the liposome and allowed the selective targeting of CD133^+^ EpCAM^+^ liver CSCs. After arriving to the CSCs, the CEP-LP@S/D liposome undergoes endocytosis to the cytoplasm, where a high concentration of glutathione (GSH) breaks its disulfide bonds, thereby degrading the liposome. This then induces a rapid release of Dox and Sal to synergistically inhibit tumor growth. Notably, this effect occurs through Dox-induced apoptosis and concurrent lysosomal iron sequestration by Sal. Interestingly, both *in vitro* and *in vivo* studies indicated that our GSH-responsive co-delivery system not only effectively enhanced CSC targeting but also eliminated the non-CSC faction, thereby exhibiting high antitumor efficacy. We believe that the smart liposome nanocarrier-based co-delivery system is a promising strategy to combat liver cancer, which may also lay the groundwork for more enhanced approaches to target other cancer types as well.

## Introduction

Hepatocellular carcinoma (HCC) is one of the most common malignant tumors in China with high mortality and incidence rates. Despite advances in diagnostic techniques and treatment approaches, most patients with advanced HCC have a poor prognosis, which may be partly attributed to a high ratio of cancer stem cells (CSCs). CSCs are a rare subset of cells that are involved in tumor maintenance, metastasis, drug resistance, and relapse (Wang et al., 2015a). Unfortunately, conventional chemotherapy and radiotherapy approaches are ineffective against CSCs and are prone to tumor recurrence, treatment failure, and ultimately death (Yarchoan et al., 2019). Salinomycin (Sal) is a potent drug that has been recently shown to selectively inhibit CSCs in various types of cancers, including liver CSCs (Gupta et al., 2009; Mai et al., 2017). However, Sal possesses unfavorable properties, such as hydrophobicity and nerve and muscle toxicity, that greatly hinder its clinical application (Wang et al., 2017). In the past decades, CD133 and EpCAM have been widely studied as stem cell markers in liver cancer (Mikhail and He, 2011). These surface markers serve not only as tools for identifying and isolating liver CSCs but also as therapeutic targets for eradicating these cells (Chan et al., 2014; Jiang et al., 2015; Saygin et al., 2019). Therefore, targeting these CSC-specific markers by optimized drug combinations would be an ideal method for overcoming the stemness of liver CSCs and ameliorating the disease (Clevers, 2011). Considering the importance of CSCs and the shortcomings of conventional anticancer therapies, dual-targeted CSC-specific delivery systems can possibly overcome this dilemma and leave an impact on the clinical setting (Dianat-Moghadam et al., 2018; Guo et al., 2019).

One way to address this issue is by using multifunctional nanoparticle (NP) systems, such as polymeric NPs, liposomes, and micelles, that can simultaneously deliver multiple therapeutic agents to induce a synergistic effect on CSCs (Zhao et al., 2014; Rao et al., 2015; Shen et al., 2015). Liposomes are an FDA-approved drug delivery system that can be loaded with both hydrophilic and hydrophobic drugs in amphiphilic lipid bilayers (Kim et al., 2015; Dianat-Moghadam et al., 2018). Peptide ligands are widely recognized as the surface modification elements in targeted-delivery therapeutic approaches (Zhang et al., 2012; Mao et al., 2015). Our previous studies have demonstrated that peptide-conjugated liposomes can facilitate drug accumulation at tumor sites and improve the anticancer effect of different drugs (Wang et al., 2019). Recently, the use of pro-drug-loaded stimuli-responsive drug delivery systems to facilitate the delivery of anticancer drugs has become a notable trend (Jia et al., 2018; Yang et al., 2018). Notably, stimuli-responsive NPs can respond to external stimuli (e.g., redox, reactive oxygen species, pH, and enzymes) to release drugs in a controlled manner (Ling et al., 2019; Liu et al., 2019). Glutathione (GSH) is a major antioxidant involved in many physiological processes that is abundant in cancer cells (Yu et al., 2019). Therefore, it is no surprise that numerous GSH-responsive nanocarriers have been developed to deliver drugs and as imaging agents for better diagnostic and therapeutic efficacy (Li et al., 2020). Nonetheless, this technology still has ways to go, as developing more efficient and smarter nanocarriers that can overcome the current challenges associated with CSCs is crucial (Shen et al., 2016; Tan et al., 2018; Reda et al., 2019).

**Scheme 1:**
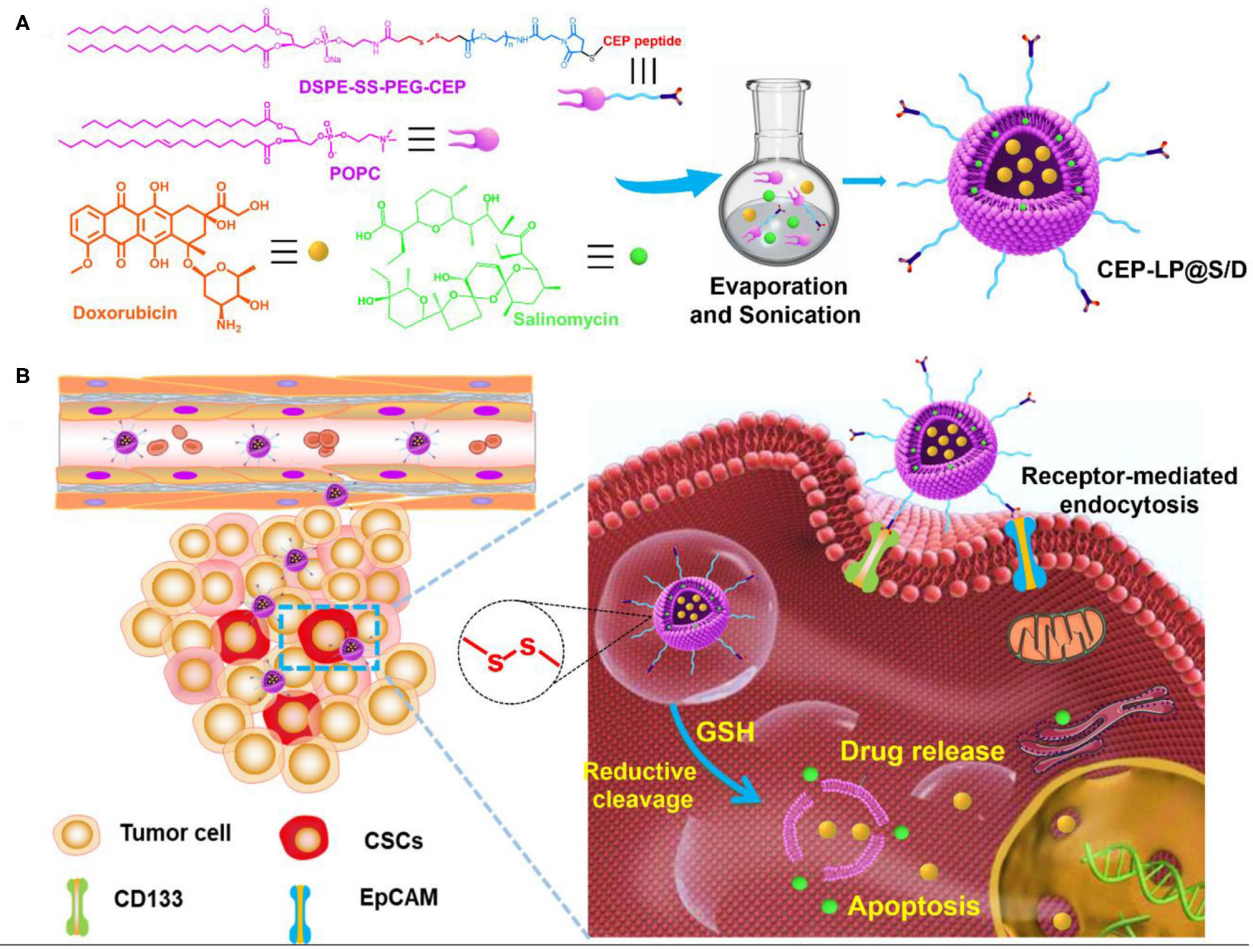
Schematic illustration of glutathione (GSH)-responsive CEP-LP@S/D liposome for controlled drug delivery. **(A)** Schematic illustration of the structure and formation of the CEP-LP@S/D liposome. **(B)** Schematic illustration of the CEP-LP@S/D *in vivo* mechanism of action and transport.

Herein, we aimed to develop a redox-responsive liposome, hereafter known as CEP-LP@S/D, that is capable of potentially targeting both liver CSCs and bulk cancer cells. The basis of this approach was the incorporation of Sal, a hydrophobic drug, into the lipid layers of the liposome and of doxorubicin (Dox), a hydrophilic drug, into the aqueous cavity of the liposome. Considering that biomarkers are heterogeneously expressed in HCC, a CD133, and EpCAM dual-targeted Y-shaped peptide ligand, CEP, was employed to decorate the surface of this liposome in order to improve both recognition and binding to CSC subpopulations. Moreover, we went further and endowed CEP-LP@S/D with GSH-responsive properties to initiate anticancer drug release in an intracellular manner once the liposomes have made contact with the tumor cells and investigated the effects both *in vitro* and *in vivo* (Scheme 1).

## Materials and Methods

### Materials

We obtained 1-palmitoyl-2-oleoyl-*sn*-glycero-3-phosphocholine (POPC) from A.V.T. Pharmaceutical Co., Ltd. (Shanghai, China), and phosphoethanolamine-*N*-[methoxy(polyethylene glycol)-2000] (DSPE-SS-PEG-2000) from Xi'an ruixi Biological Technology Co., Ltd. (Xi'an, China). Meanwhile, we acquired 1,1′-dioctadecyl-3,3,3′,3′-tetramethylindotricarbocyanine iodide (DiR), LysoTracker Green, and 3-(4,5)-dimethylthiahiazol(-z-y1)-3,5-di-phenytetrazoliumbromide (MTT) from Sigma-Aldrich. We purchased Dulbecco's modified Eagle's medium (DMEM) and fetal bovine serum (FBS) from Gibco (USA) and Sal from Selleck Chemicals (USA). Moreover, we obtained FITC-EpCAM and PE-CD133 antibodies from BioLegend (Shanghai, China).

### Preparation and Characterization of CEP-Liposome NPs

In our previous work, we screened EP1 (YEVHTYYLD) and CY (CYIVFYDSPLE) as specific peptides toward EpCAM and CD133, respectively, using a high-throughput library (Wang et al., 2015b). These two peptide ligands were connected via a GG linker, leading to the formation of Y-shape peptides (CEP), which was synthesized by the Fmoc solid-phase synthesis technique. The CEP was then covalently conjugated to DSPE-SS-PEG2000-Mal by adding thiol groups and maleimide (Michael addition). Liposomes were fabricated according to the literature (Guo et al., 2019). Briefly, POPC/DSPE-SS-PEG2000-CEP (molar ratio 19:1) was mixed and dissolved in chloroform/methanol (v/v, 2:1). Next, a certain amount of Sal and Dox (mole ratio 1:1.5) was dissolved in 1 ml of methanol at room temperature and mixed with the lipid solution. The mixed solvent was then dried by rotary evaporation at 45°C to form a lipid film. After that, the dried film was hydrated with 5 ml of phosphate-buffered saline (PBS; pH 7.4) for 30 min and sonicated for 10 min. Finally, the prepared drug-loaded liposome, CEP-LP@S/D, was filtered through a 200-nm membrane filter to remove any precipitates. As for our *in vivo* imaging studies, we used DiR as the fluorescent probe; thus, DiR-loaded CEP-LP@S/D liposomes were prepared following the same procedures as above. The morphology and size of CEP-LP@S/D were measured by transmission electron microscopy (TEM), while their hydrodynamic size and polydispersity (PDI) were further measured in aqueous solutions by dynamic light scattering (DLS).

### *In vitro* Encapsulation and Release Profiles

The amount of Sal encapsulated in CEP-LP@S/D was detected at 392 nm by high-performance liquid chromatography (HPLC), and Dox fluorescence (λ_Ex_: 480 nm; λ_Em_: 590 nm) was measured using a fluorescence spectrometer. The drug encapsulation efficiency (EE) and the loading efficiency (LE) were then calculated according to the equations established by Zhang et al. (2012). Next, *in vitro* drug release from the liposomes was investigated by using a dialysis method. In short, the CEP-LP@S/D liposomes were dispersed in 1 ml of PBS (pH 7.4) and transferred to a dialysis device (MWCO: 10 kDa); they were then immersed in a GSH-containing PBS solution at 37°C and gently stirred. The Dox or Sal content released in the medium was determined by fluorescence spectrometry and HPLC at different time points as described above.

### Cytotoxicity and Mammosphere Formation Assays

To evaluate the efficacy of the combination therapy *in vitro*, Huh-7 cells, or human HCC cells, were seeded in 96-well plates (5 × 10^3^ cells per well) at 37°C for 12 h. After that, the cells were incubated with various concentrations of Dox, Sal, LP@S/D, or CEP-LP@S/D for 48 h, and cell viability was assessed by the MTT assay.

We further performed the mammosphere formation assay to assess treatment-induced changes in the stemness of these cells, that is, the CSC self-renewal ability. Thus, EpCAM^+^ CD133^+^ Huh-7 single-cell suspensions were sorted and seeded in ultralow-attachment six-well plates at a density of 5 × 10^3^ cells per well. Then, various concentrations of free Sal, LP@S/D, or CEP-LP@S/D were added, and the cells were incubated at 37°C for 24 h. Thereafter, the cells were washed with 1 × BS and cultured in a serum-free DMEM/F12 medium, supplemented with 1 × B27 (Invitrogen), 20-ng/ml recombinant human epidermal growth factor (PeproTech), and 20-ng/ml basic fibroblast growth factor (PeproTech). After that, the cells were cultured in a 5% CO_2_ incubator at 37°C for 7 days. The formed mammospheres in each treatment condition were counted and visualized under an optical microscope. Furthermore, to determine whether CEP-LP@S/D could induce a durable mammosphere inhibitory response, the primary mammospheres were trypsinized, prepared into single-cell suspensions, and then cultured in ultralow-adherent six-well plates as previously described. After 5 days, the number and morphology of the secondary mammospheres in each treatment condition were monitored and imaged under a microscope. The saline-treated group was considered as the control.

### Cellular Uptake and Localization

The cellular internalization of LP@S/D and CEP-LP@S/D was studied by confocal laser scanning microscopy (CLSM). Huh-7 cells were cultured into glass-bottom dishes and incubated for 24 h, after which the cells were incubated with either CEP-LP@S/D or LP@S/D (20 μg/ml) as described above. After 4–8 h of incubation, the cells were washed and stained with 4′,6-diamidino-2-phenylindole (DAPI), a cell-permeant fluorescent nuclear dye, for 15 min and then rinsed with PBS. Finally, the cells were examined using a Zeiss 710 confocal microscope. In addition, we investigated the intracellular distribution of CEP-LP@S/D in the dissociated mammosphere cells via CLSM. Briefly, CD133^+^ EpCAM^+^ Huh-7 mammosphere cells were seeded into 24-well plates in a DMEM/F12 medium. After 24 h of incubation, they were treated with CEP-LP@S/D or LP@S/D at a concentration of 50 μg/ml for 8 h. Finally, the cells were collected and stained, as described above, and visualized using CLSM.

### The Expression of Stemness-Associated Genes

To assess the stemness of CD133^+^ EpCAM^+^ Huh-7 tumorspheres, we extracted total RNA from the tumorspheres using TRizol (Invitrogen Inc., USA). Total RNA was converted into cDNA by using the PrimeScript™ RT Reagent Kit (Takara, China). Next, SYBR Green PCR Master Mix was added to the obtained cDNA, which was then quantified using the ABI PRISM 7,700 real-time polymerase chain reaction (PCR) platform. The mRNA expression levels of *Sox-2, Oct-4, ABCG2*, and *CD133* were normalized against that of *GAPDH* and those of PBS-treated cells.

### *In vivo* Imaging to Evaluate the Biodistribution of CEP-LP@S/D

Tumor-bearing mice were intravenously injected with (i) PBS, (ii) LP@S/D, or (iii) CEP-LP@S/D. After 2 h, the mice were imaged using an *in vivo* imaging system (IVIS). DiR-containing CEP-LP@S/D were detected by the appearance of a fluorescent signal at 748/780 nm (λ_Ex_/λ_Em_), and images were acquired using the IVIS. At the end point of the experiment, the mice were euthanized, and their major organs were harvested and analyzed to assess the biodistribution of CEP-LP@S/D in each organ.

### *In vivo* Antitumor Efficacy

For *in vivo* studies, BALB/C mice (6 weeks old) were subcutaneously (s.c.) injected with 1 × 10^6^ sorted CD133^+^ EpCAM^+^ Huh-7 liver CSCs in their left flanks. When the tumor volume reached about 60–80 mm^3^, the mice were randomly distributed into four groups (*n* = 3), and each group received one of the following treatments: PBS control, Sal, LP@S/D, or CEP-LP@S/D; all the formulations were administered via tail vein injection (6 mg/kg) every other day. The body weight of the mice was monitored every 3 days, and tumor volumes were calculated using the following formula: (width^2^ × length)/2; measurements were obtained using a vernier caliper every other day. The mice were sacrificed to harvest their tumors and main organs for further histological examination, namely, hematoxylin–eosin (H&E) staining and terminal deoxynucleotidyl transferase-mediated dUTP nick end labeling (TUNEL) analysis according to the manufacturer's instructions. In addition, we analyzed the CSC fraction in the tumor mass tissues to assess the effectiveness of the treatments in targeting CSCs and associated stemness features; in short, the tumor tissues were cut into small pieces and the extracted RNA was analyzed by real-time quantitative PCR (qPCR) as described above.

## Results and Discussion

### Characterization of Drug-Loaded CEP-LP@S/D Liposomes

**Figure 1:**
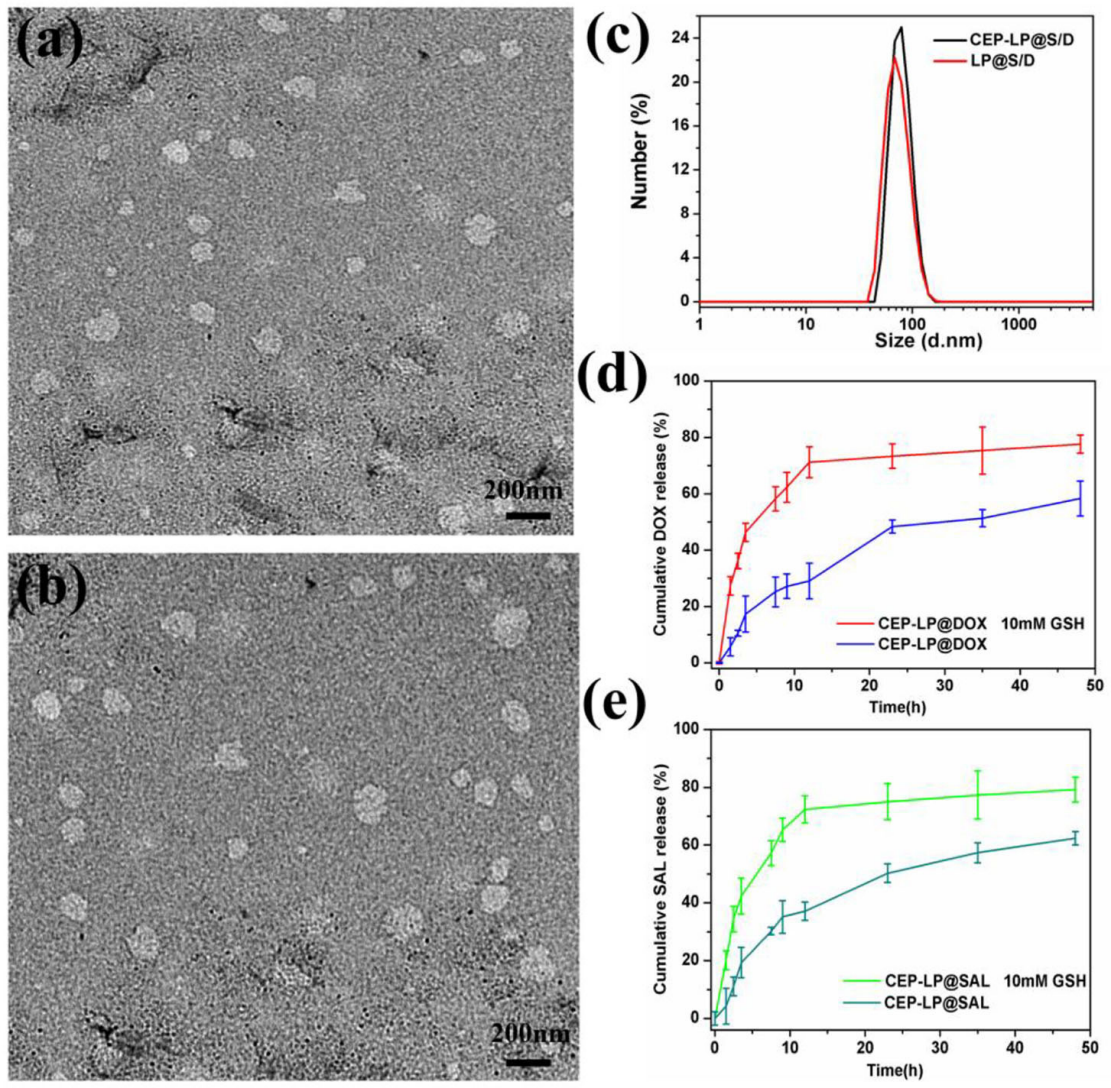
Characterization of CEP-LP@S/D. **(a)** Transmission electron microscopy (TEM) image of LP@S/D. **(b)** TEM image of CEP-LP@S/D. **(c)** Hydrodynamic diameters of CEP-LP@S/D and LP@S/D measured by dynamic light scattering (DLS). **(d)** The *in vitro* release profiles of doxorubicin (Dox) in phosphate-buffered saline (PBS; pH 7.4) in the presence or absence of 10 mM glutathione (GSH). **(e)** The *in vitro* release profiles of salinomycin (Sal) in PBS (pH 7.4) in the presence or absence of 10 mM GSH.

Herein, we introduce a Y-shaped surface modification-based material, CEP-DSPE-SS-PEG, that bears two targeting peptides EpCAM and CD133, one in each head (Supplementary Figure 1). CEP-DSPE-SS-PEG was synthesized through covalent conjugation between the thiolated peptide CY-EP1 and DSPE-SS-PEG2000-Mal as confirmed by matrix-assisted laser desorption/ionization time-of-flight mass spectrometry (MALDI-TOF-MS) (Supplementary Figure 2; Belhadj et al., 2017). The anticancer drug Dox and the newly categorized anticancer agent Sal were loaded into CEP-LP@S/D and LP@S/D via the solvent evaporation method. The measurements showed that CEP-LP@S/D and LP@S/D were within a similar size range diameter-wise (Figures 1a,b). The average particle size for all liposome systems was around 115 nm with a small PDI value of <0.28 and good dispersion (Figure 1c). Notably, the particle size was not significantly affected by the CEP peptide modification. Moreover, the TEM images (Supplementary Figure 3) illustrated that the nanostructure of CEP-LP@S/D was disrupted in a GSH-rich surrounding after 8 h. To further confirm the capability of CEP-LP@S/D to simultaneously deliver two drugs, we tested the encapsulation efficiencies of Sal and Dox in CEP-LP@S/D using HPLC (for Sal) and a fluorescence spectrometer (for Dox). The liposomal EE for both Dox and Sal was higher than 86%. Furthermore, the drug-loading capacities of CEP-LP@S/D and LP@S/D were determined to be 2.4 and 2.2%, respectively. Additionally, the drug-release behaviors of CEP-LP@S/D were investigated with or without GSH (10 mM) in PBS (pH = 7.4) at 37°C. As shown in Figures 1d,e, CEP-LP@S/D exhibited the ability of controlled release, as only about 37 and 29% of the Sal and Dox, respectively, were released from the drug-loaded CEP-LP@S/D after 12 h without the presence of GSH. However, a markedly enhanced drug release rate was detected in the presence of GSH (10 mM). Evidently, the GSH could effectively break the disulfide bonds of the CEP-LP@S/D lipid layers, thereby inducing the disassociation of the liposomal nanostructure; notably, more than 77.6 ± 1.4% of the loaded Dox and 79.2 ± 1.7% of the loaded Sal were released within 48 h in this case. We believe that the accelerated rate of Dox and Sal release by CEP-LP@S/D in the presence of GSH may be attributed to intracellular reducing environment-induced drug release.

### Cellular Uptake and Localization

**Figure 2:**
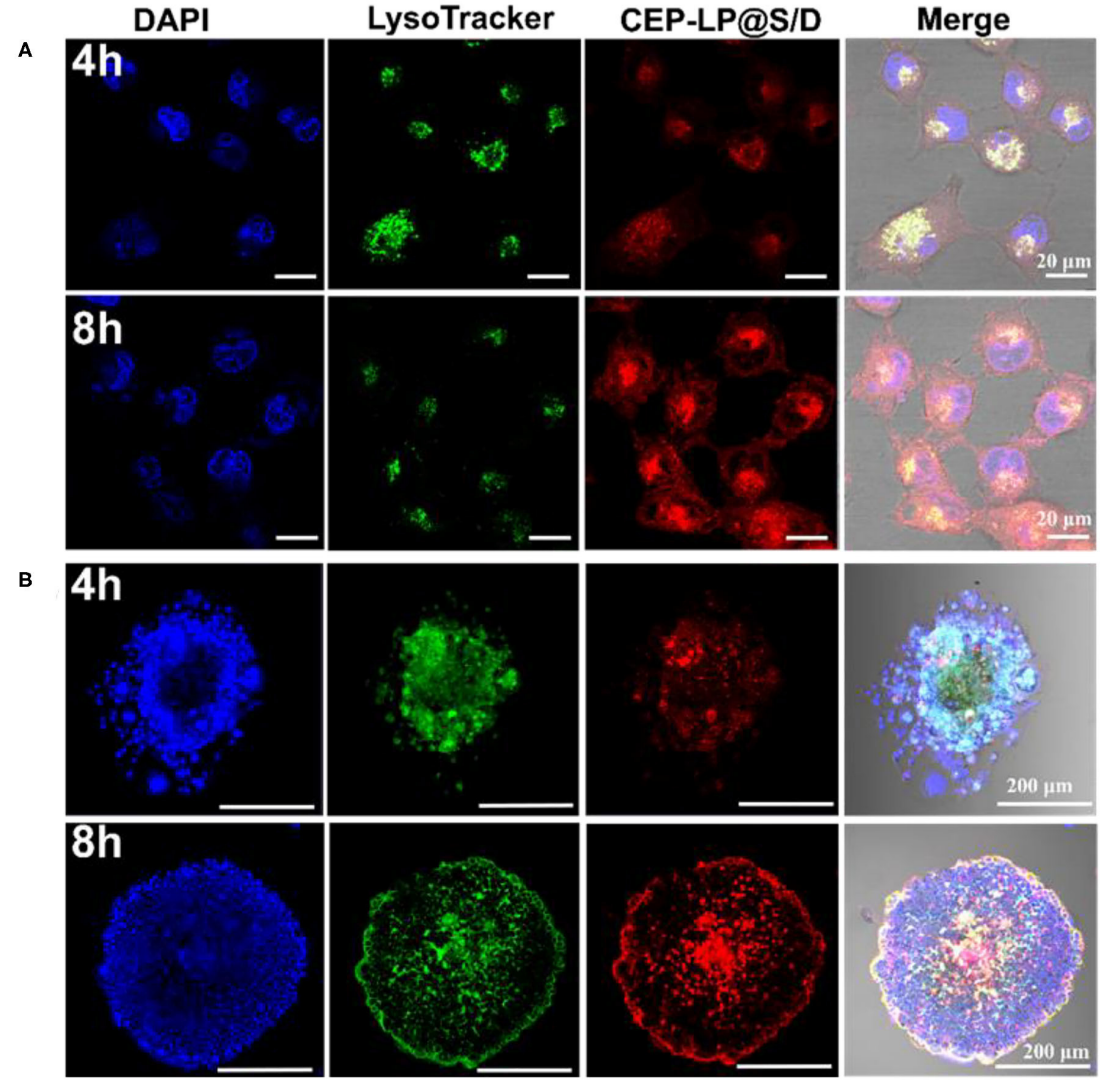
CEP-LP@S/D uptake by confocal laser scanning microscopy (CLSM) imaging. **(A)** CLSM images of Huh-7 cells incubated with CEP-LP@S/D for 4 and 8 h. Three fluorescent dyes were used to evaluate CEP-LP@S/D uptake; the red signal represents doxorubicin (Dox), the green signal indicates lysosomes (LysoTracker Green), and the blue signal (4′,6-diamidino-2-phenylindole, DAPI) reveals the nuclei. **(B)** CLSM images of tumorsphere cells incubated with CEP-LP@S/D for 4 and 8 h.

The intracellular accumulation and distribution of CEP-LP@S/D in Huh7 cells were studied using confocal microscopy. As shown in Figure 2A, after 4 h incubation, CEP-LP@S/D-treated cells displayed more fluorescent signals in their cytoplasms than did their LP@S/D-treated counterparts (Supplementary Figure 4). As time went by, red fluorescent signals indicated that Dox could diffuse into cell nuclei, indicating that the EpCAM and CD133 peptides allowed for receptor-mediated endocytosis, thereby increasing the CEP-LP@S/D uptake and internalization by CSCs. In contrast, CEP-LP@S/D-treated 293T cells showed weak red fluorescence (Supplementary Figure 5). On another note, these results strongly demonstrate that CEP-LP@S/D could respond to the intracellular redox environment, leading to the disruption of the disulfide bonds in the lipid membranes, which consequently enabled the highly hydrophobic Dox to readily penetrate into the cells and, thus, exhibit greater cellular accumulation. Additionally, to evaluate the CSC-targeted effect, we established CD133^+^EpCAM^+^ tumorspheres as CSC models; of course, we confirmed the stemness of these *in vitro* spheroids beforehand by analyzing the widely known stem cell markers *Oct-4, Sox-2*, and *CD133*, which were all overexpressed in these spheres (Supplementary Figure 6). As shown in Figure 2B, fluorescently labeled CEP-LP@S/D (red) were obviously notable in the CSC tumorspheres and had a high intensity. Furthermore, after 8 h incubation, the red fluorescence was significantly higher and could be detected in the nuclei. These results indicate that the Y-shaped peptide modification not only led to effective targeting of CSCs but could also facilitate the permeability of liposomal NPs to the inner part of the tumorspheres. Notably, CEP-LP@S/D was efficiently accumulated in the tumors and could effectively recognize and eradicate the CSC faction.

### Cytotoxicity and CSC Elimination *in vitro*

**Figure 3:**
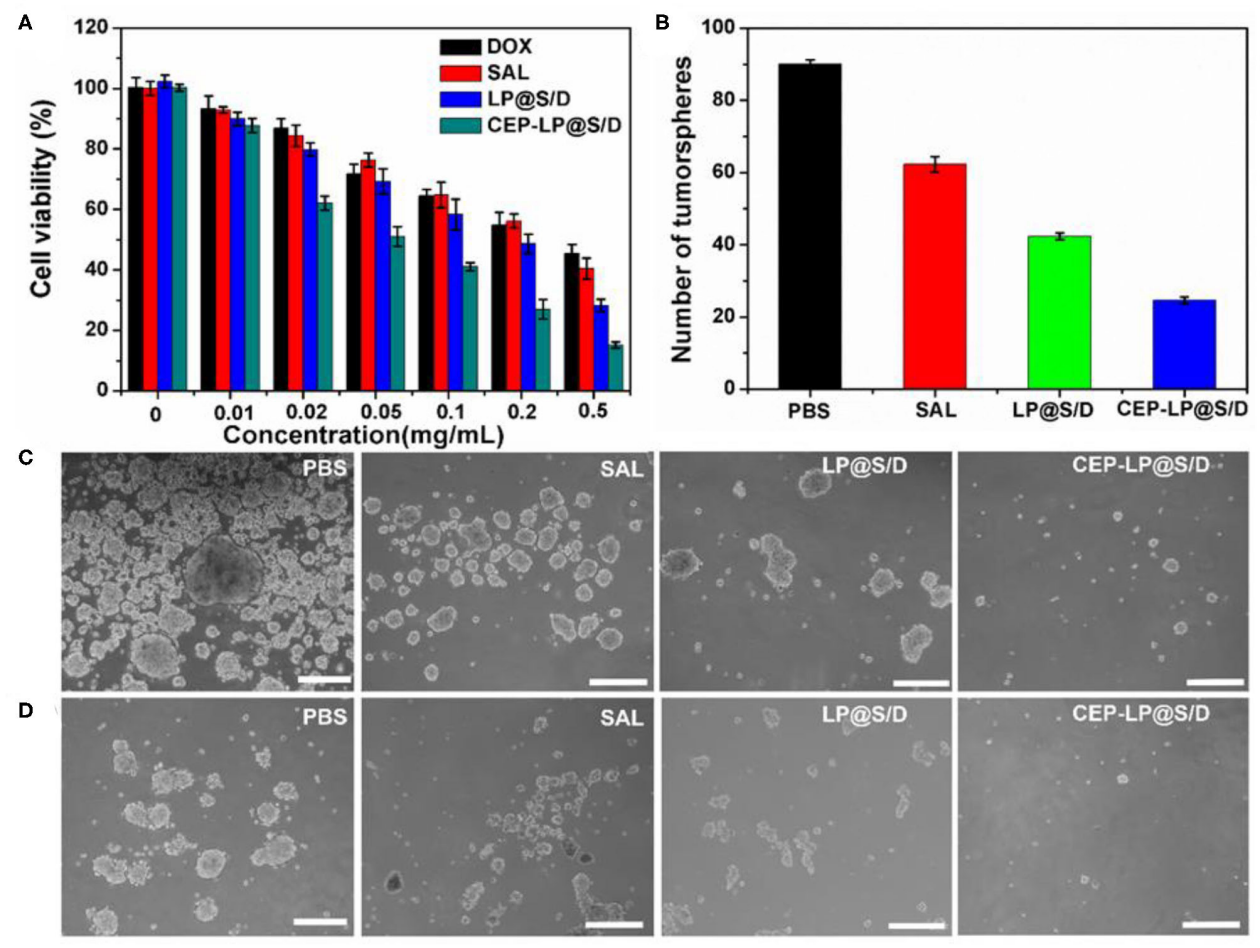
The inhibitory effect of various groups on the tumorsphere-forming ability of Huh-7 cells. **(A)** Viability of Huh-7 cells treated with free doxorubicin (Dox), Sal, LP@S/D, or CEP-LP@S/D for 48 h. **(B)** Quantification of tumorsphere formation efficiency in Huh-7 cells treated with Sal, LP@S/D, or CEP-LP@S/D. **(C)** Images of tumorsphere formation of untreated Huh-7 cells (phosphate-buffered saline, PBS) and those treated with salinomycin (Sal), LP@S/D, or CEP-LP@S/D for 5 days. **(D)** Images of second-generation tumorsphere formation (secondary formation after dissociation of initial spheres) under PBS, Sal, LP@S/D, or CEP-LP@S/D treatment. Scale bar = 300 μm.

The cytotoxicity of the CEP-LP@S/D liposomes was evaluated by the MTT assay. Huh-7 cells were treated with Dox, Sal, LP@S/D, or CEP-LP@S/D at concentrations ranging from 0.01 to 0.5 mg/ml for 48 h. As shown in Figure 3A, CEP-LP@S/D exhibited higher cytotoxicity than did LP@S/D and the free drugs; notably, both Dox and Sal exhibited low cytotoxicity alone. This likely suggests that the dual-targeted peptide-mediated cellular uptake and GSH-triggered intracellular reductive cleavage improved the release of the loaded drugs. As expected, the tumorsphere formation assay revealed that CEP-LP@S/D induced a dramatic decrease in the number of formed tumorspheres than did the LP@S/D- and Sal-treated groups (Figure 3B). Moreover, as shown in Figure 3C, PBS (the vehicle) did not affect the tumorsphere-forming ability of Huh-7 cells. Interestingly, although the percentage of formed tumorspheres was marginally reduced by Sal and LP@S/D treatment, the size of the spheroids remained largely unaltered. However, the size of the tumorspheres in the CEP-LP@S/D treatment group was notably the smallest among all the groups, which suggests synergistic effects between the two drugs. To determine whether CEP-LP@S/D could induce a durable tumorsphere inhibitory response, CEP-LP@S/D-treated primary tumorspheres were dissociated into single-cell suspensions, and their propensity to form secondary tumorspheres was assessed (Figure 3D). It was shown that the CEP-LP@S/D group had a maximum of 89.2% inhibition rate. Once again, single-cell suspensions of Sal- and LP@S/D-treated primary tumorspheres produced more secondary tumorspheres than did those of the free Sal-treated group; however, their CEP-LP@S/D-treated counterparts exhibited non-clonogenic properties (Suntharalingam et al., 2014). It is worth mentioning that tumorsphere cells are much more resistant to free Sal, a problem that can be effectively overcome by using the liposomal construct prepared in this study. Taken together, these data show that CEP-LP@S/D inhibits the self-renewal of Huh-7 tumorspheres by eliminating the CSC population (CD133- and EpCAM-positive) and that this effect is maintained upon serial passages. Therefore, these results support the use of CEP-LP@S/D for the efficient internalization of drugs into CSC tumorspheres and preferentially inhibiting the proliferation of CSCs.

### Tumor Accumulation of CEP-LP@S/D *in vivo*

**Figure 4:**
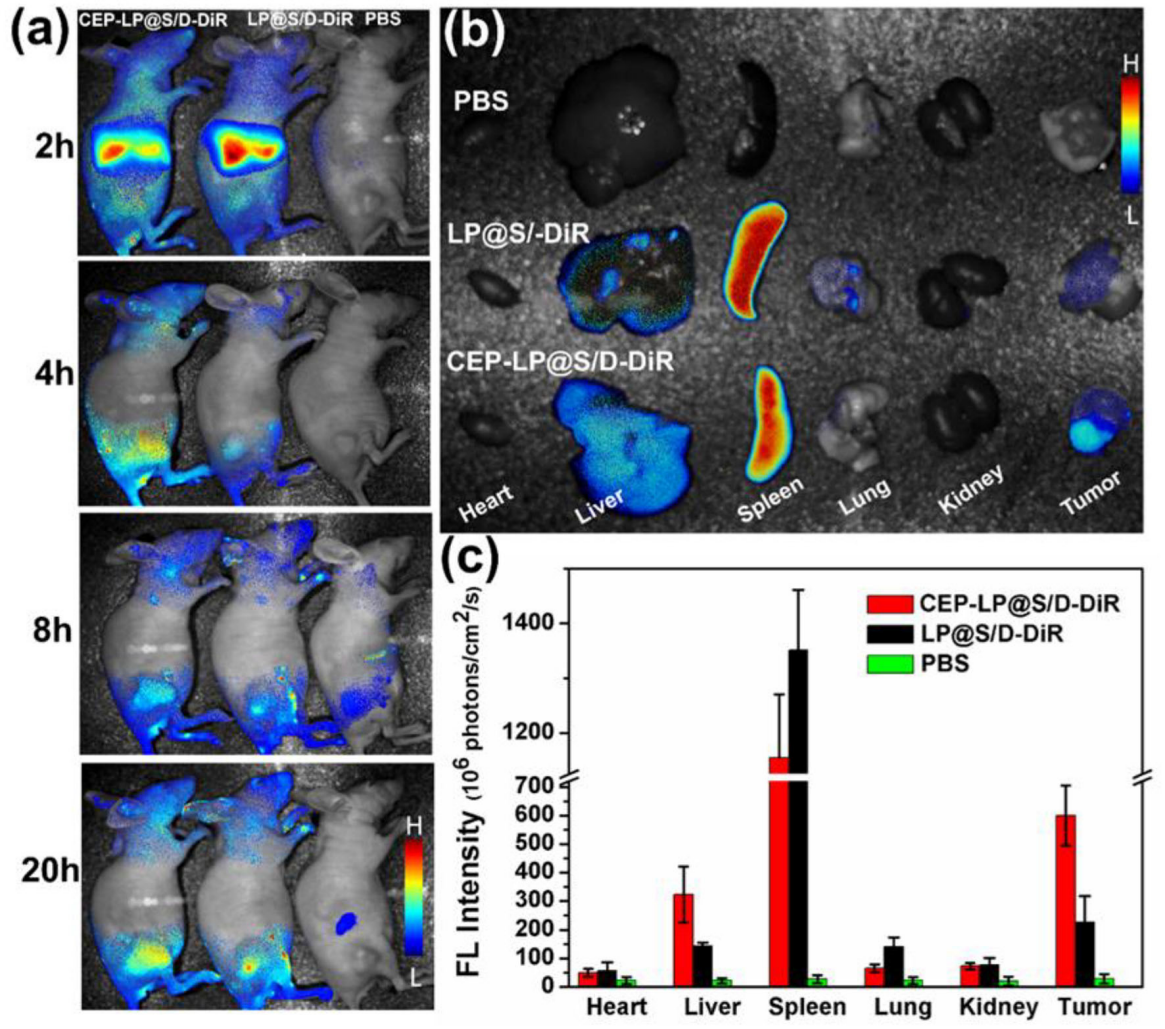
The *in vivo* imaging distribution of CEP-LP@S/D. **(a)** *In vivo* image of Huh-7 xenograft tumor-bearing nude mice at different time points after injection with DiR-loaded CEP-LP@S/D liposomes. **(b)** *Ex vivo* image of tumors and main organs 24 h post injection of the different formulations. **(c)** The quantified biodistribution of CEP-LP@S/D in the major organs 24 h post injection.

The ability of CEP-LP@S/D to home toward tumors was examined by the IVIS. The CEP-LP@S/D liposomes were loaded with DiR dye prior to being injected into tumor-bearing mice. As shown in Figure 4a, DiR fluorescent signals were obviously detected in the tumor sites 2 h after the injection and gradually increased with time. Notably, CEP-LP@S/D-DiR signals peaked after 20 h and gradually disappeared after the 24-h mark; moreover, they were markedly higher than those of LP@S/D-DiR. These findings clearly demonstrate that the CEP-LP@S/D liposomes remain in the tumors for a satisfactory amount of time (good retention time) and prove that they can efficiently and effectively target the tumor site in both passive and active manners. These characteristics are possible due to the Y-shaped CD133 and EpCAM ligand surface modification, which led to the enhanced permeability and retention (EPR) effect. The *ex vivo* histological analysis (biodistribution study) showed that the fluorescent CEP-LP@S/D-DiR signals were mostly detected in the tumors and spleens of the mice (Figure 4b). However, these signals were weaker in other organs, which exhibited only background or moderate signals, suggesting the preferable accumulation of the liposome in tumor tissues (Figure 4c). This result indicates that the CEP-LP@S/D liposomes favorably accumulate in the tumors, where they have a notable penetration capacity that could significantly increase the possibility of CEP-LP@S/D to eradicate CSCs *in vivo*.

### *In vivo* Antitumor Efficacy of CEP-LP@S/D

**Figure 5:**
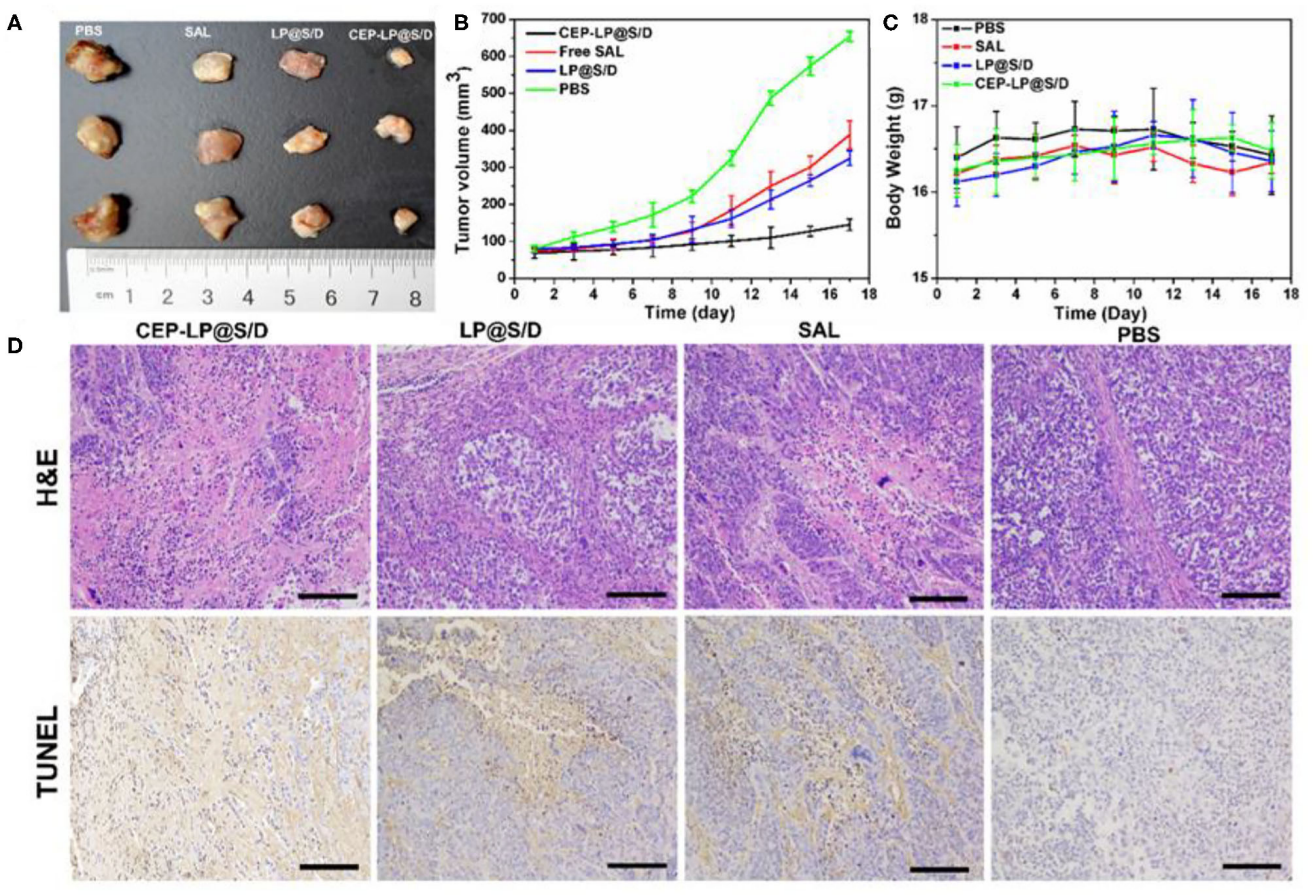
*In vivo* antitumor activity of the different formulations on Huh-7-bearing nude mice. **(A)** The photographs of tumors in the differently treated Huh-7-bearing nude mice 18 days after tumor excision and experiment end point. **(B)** A curve representing the tumor growth of the differently treated mice. **(C)** The weight of the mice during the treatment. **(D)** Hematoxylin–eosin (HandE) and terminal deoxynucleotidyl transferase-mediated dUTP nick end labeling (TUNEL) analyses of tumor tissues after treatment with various formulations. Scale bar = 0.1 cm.

To further verify whether CEP-LP@S/D could facilitate the accumulation of Sal and Dox in the xenograft-induced tumors, different formulations, with equivalent Dox and Sal doses of 6 mg/kg, were administered via tail vein injection every other day. As shown in Figures 5A,B, CEP-LP@S/D liposomes exhibited the best antitumor effect than did the other two groups. However, free Sal also showed a slight tumor inhibitory effect at the same time points, although this variation was not statistically significant. This suggests that the Y-shaped peptidic construct led to more extensive intracellular delivery via receptor-mediated targeting of EpCAM^+^CD133^+^ liver CSCs. In accordance with our *in vitro* results, CEP-LP@S/D disulfide bond breakage in response to elevated GSH levels in the cytosol explains the enhanced release of Dox and Sal, which in turn induce a synergistic cytotoxic effect against bulk tumor cells and CSCs. It is worth noting that none of the mice had any noticeable weight change in any of the treatment groups, suggesting there was no obvious systemic toxicity from CEP-LP@S/D (Figure 5C). A number of studies have indicated that *Sox-2* and *Oct-4* represent strong stemness characteristics that are crucial for the tumor initiation and self-renewal of CSCs (Ma et al., 2010). As shown in Supplementary Figure 7, the expression level of *Sox-2* was markedly suppressed by CEP-LP@S/D. Moreover, CEP-LP@S/D treatment also induced significant downregulation of other stemness-associated (*CD133, Oct-4*, and *Sox-2*) and drug efflux (*ABCG2*) genes than did the free Sal-treated group. This indicates that the CEP-LP@S/D liposome was capable of downregulating stemness-associated genes and synergistically enhancing drug cytotoxicity toward CSCs. We went further to assess the antitumor efficacy as well as the potential side effects; histological analysis of tumor slides demonstrated that CEP-LP@S/D was the most effective in inhibiting cell proliferation and inducing cell apoptosis, with only a few side effects (Figure 5D). In contrast, free Sal and LP@S/D did not significantly inhibit cell proliferation and showed a comparable effect to that of PBS, as demonstrated by TUNEL. Apart from these, H&E staining indicated that there is no obvious toxicity in the major organs, including the heart, liver, spleen, lung, and kidney (Supplementary Figure 8). As shown in Supplementary Figure 9, CEP-LP@S/D significantly improved the survival rate of the mice compared to other groups. Taken together, our results indicate that co-delivery of Dox and Sal via CEP-LP@S/D induced a significant synergistic anticancer effect as it combined the ability of Dox to eliminate bulk cancer cells with that of Sal to suppress the CSC population and went further to enhance their absorption and targeting abilities.

## Conclusions

To reiterate, in this study, we successfully developed a new redox-responsive liposomal platform for targeted anticancer drug delivery that allows the synergistic amelioration of liver cancer. Our results indicate that the CEP-LP@S/D co-delivery system could enhance the accumulation of drugs in the tumor tissues and target CSCs via the specific peptide recognition receptors CD133 and EpCAM, which are over-expressed on these cells. After cellular uptake, the high concentration of GSH in the cytoplasm is sufficient to break the disulfide bonds in the structure of these liposomes, thereby inducing the fast release of Dox and Sal and leading to the synergistic inhibition of tumor growth and reduction in CSC stemness. We believe that this co-delivery nano-platform could be used as an effective tool for delivering combinatorial therapeutics to synergistically inhibit liver CSCs and tumor cells. This study provides a new perspective for designing specifically responsive drug delivery systems to target CSCs and may lay the groundwork for similar approaches customized for other cancer types.

## Data Availability Statement

The original contributions presented in the study are included in the article/Supplementary Material, further inquiries can be directed to the corresponding authors.

## Ethics Statement

The animal study was reviewed and approved by Peking University Animal ethics Committee.

## Author Contributions

ZW performed the experiments and wrote the manuscript. WL, MS, and YZ helped to perform *in vitro* experiments. LF helped to characterize materials and incubate cells. ZH revised the manuscript and supervised all the works. All authors contributed to the article and approved the submitted version.

## Conflict of Interest

The authors declare that the research was conducted in the absence of any commercial or financial relationships that could be construed as a potential conflict of interest. The reviewer LJ declared a shared affiliation with authors MS, WL, LF, and ZH to the handling editor at the time of review.
