# Supplementary material for: A Novel CD133- and EpCAM-Targeted Liposome With Redox-Responsive Properties Capable of Synergistically Eliminating Liver Cancer Stem Cells
Source: Front Chem. 2020 Aug 11;8:649. doi: 10.3389/fchem.2020.00649 (PMC7431664; doi:10.3389/fchem.2020.00649)
Supplement: Supplementary file 1 [file Data_Sheet_1.docx]

Supplementary Material


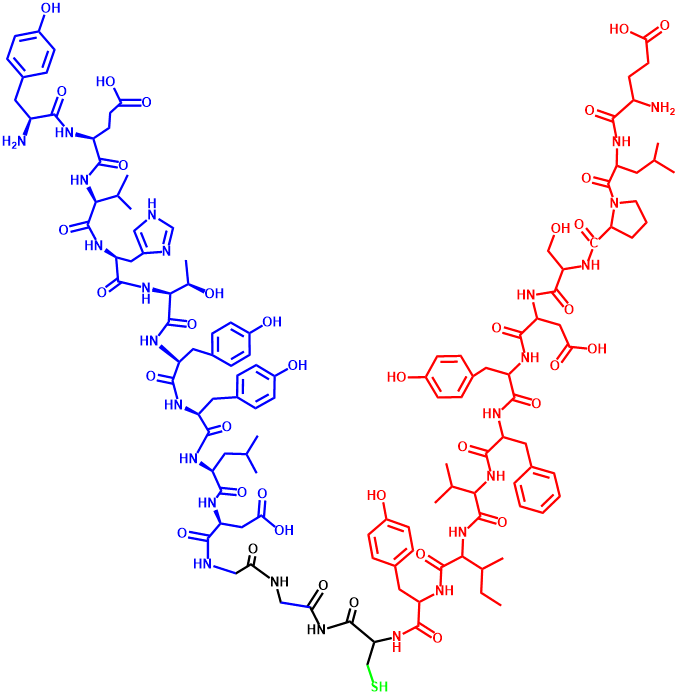


**Supplementary Figure 1**. Chemical structure of the CEP peptide.

##



**Supplementary Figure 2.** MALDI-TOF-MS analysis of the conjugation of CEP with DSPE-SS-PEG2000-MAL.


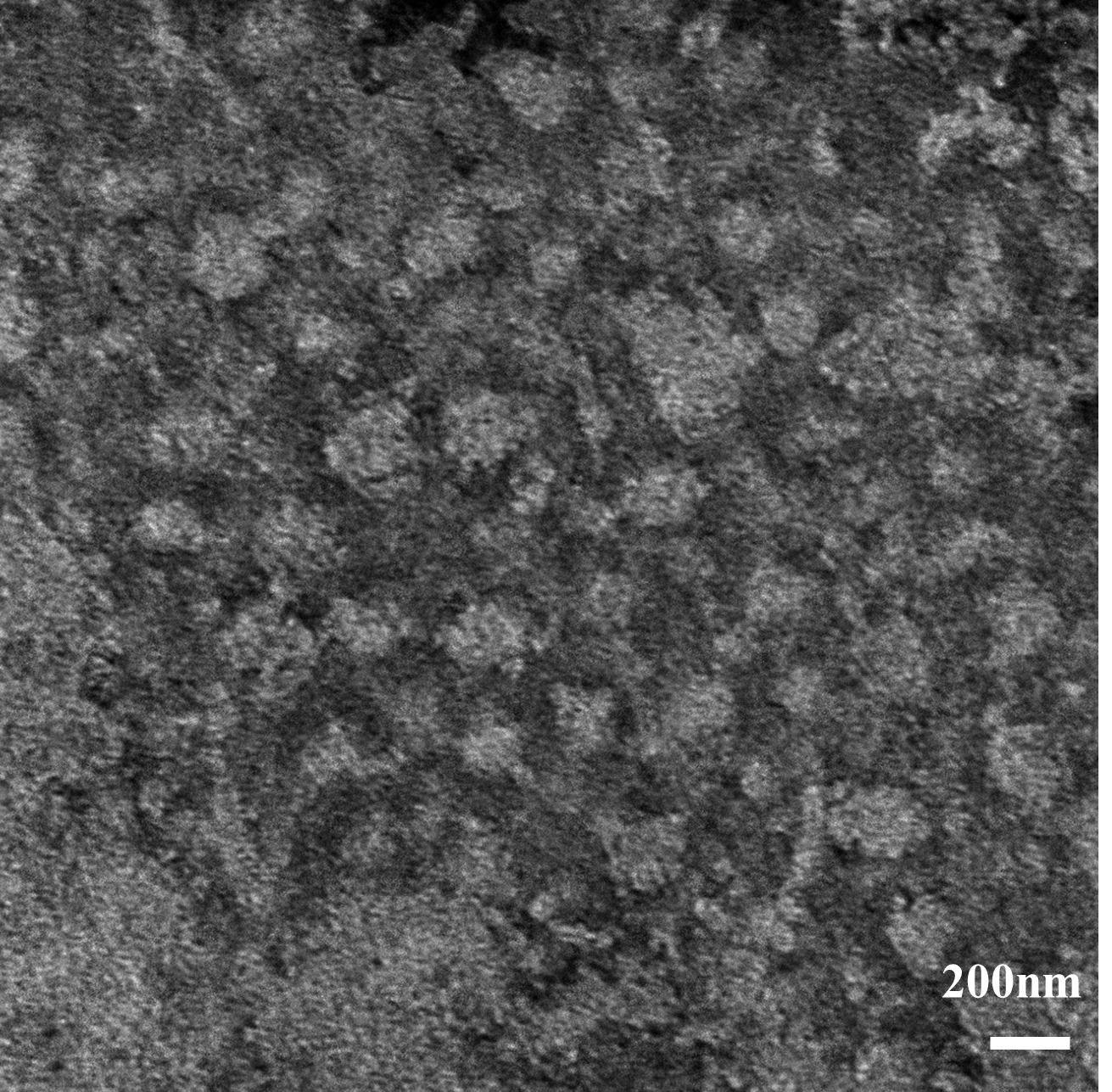


**Supplementary Figure 3.** TEM Morphology of the CEP-LP@S/D incubated in the PBS solution with 10 mM GSH for 8 h.

**
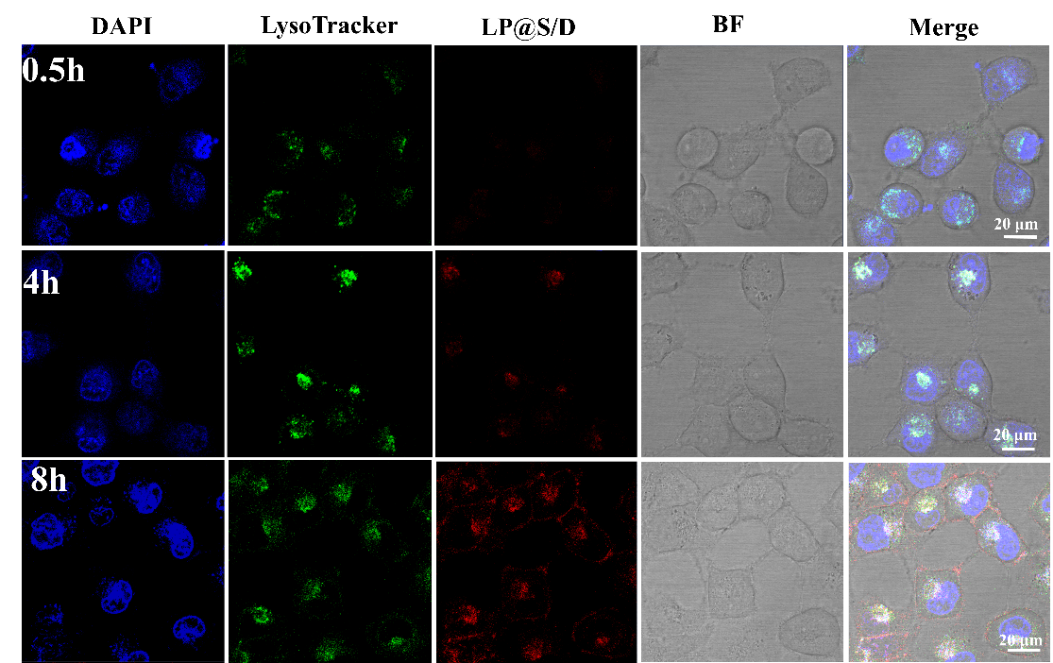
**

**Supplementary Figure 4.** Confocal images of time-dependent uptake of LP@S/D in Huh-7 cells. Scale bar is 20 μm.


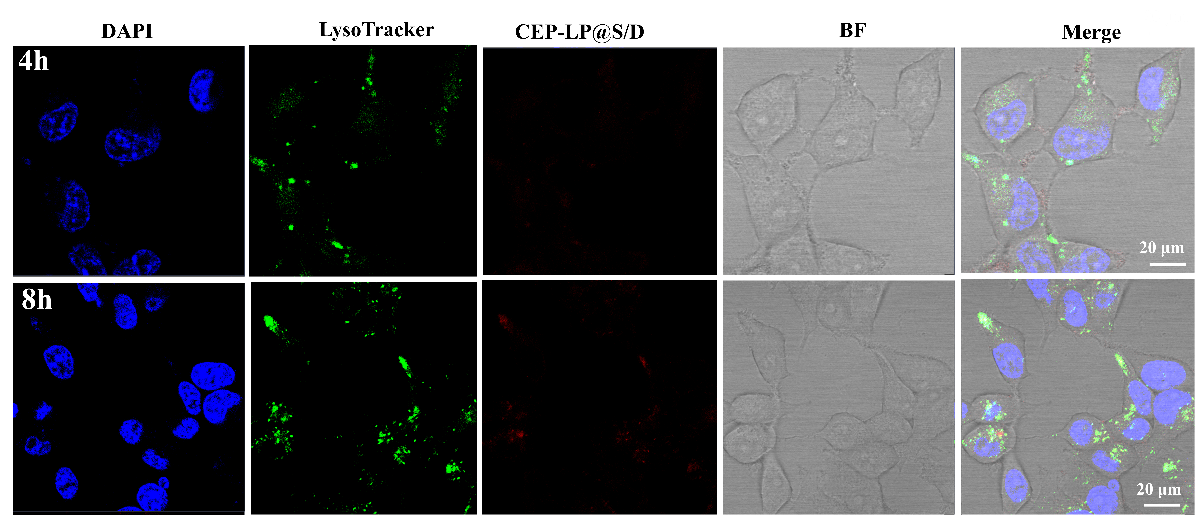


**Supplementary Figure 5.** Confocal images of CEP-LP@S/D with negative control cells 293T. Scale bar is 20 μm.

**

**

**Supplementary Figure 6.** The expression of stemness-associated genes in CD133^+^ EpCAM^+^ HCC mammosphere cells. Data are shown as means ± s.d. (n =3).

**

**

**Supplementary Figure 7.** The change of stemness-associated genes in tumor after 18 days of treatment with different formulations. The mRNA expression level of each group was normalized by GAPDH. Data represented as mean ± SD (n = 3).


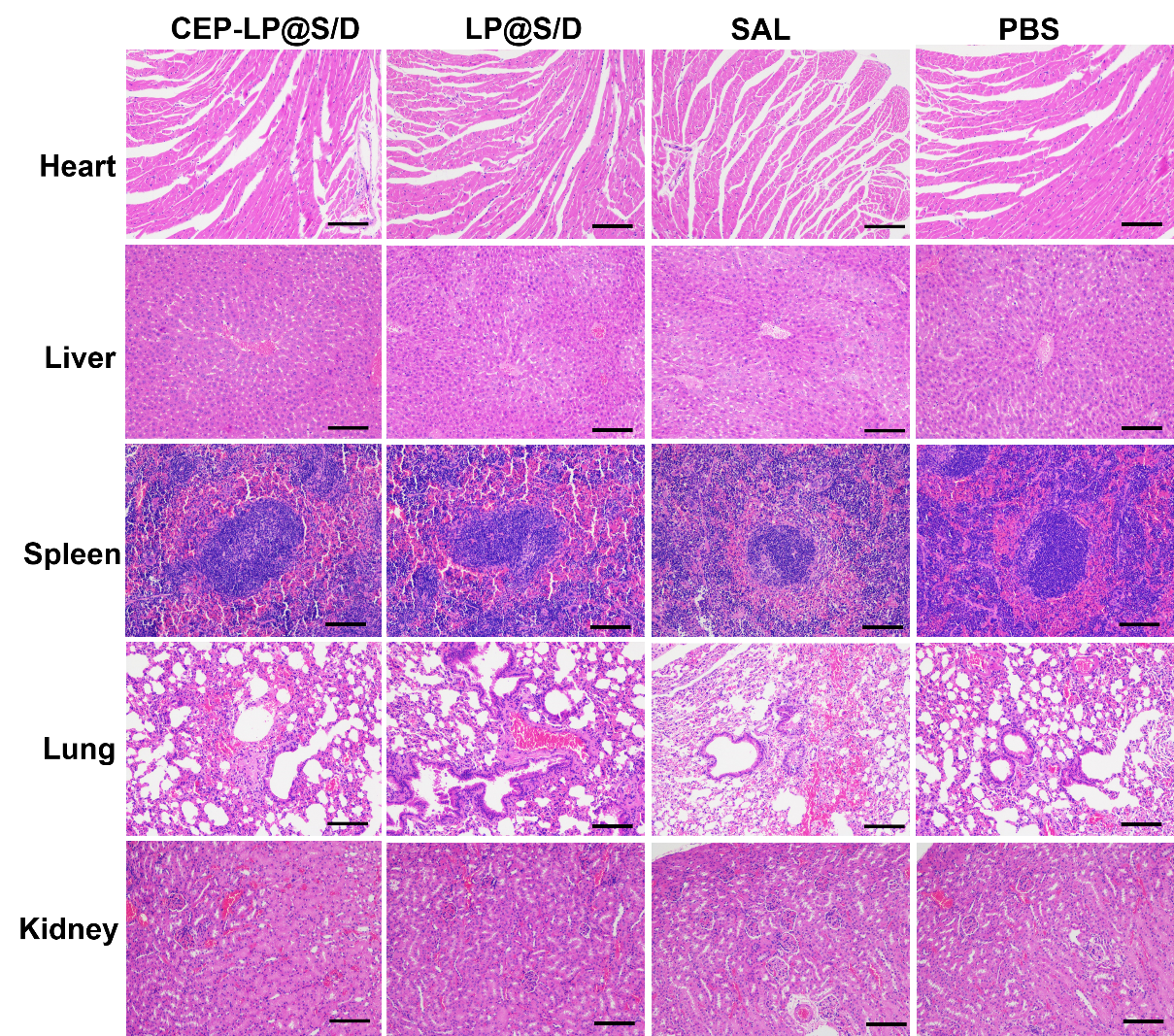


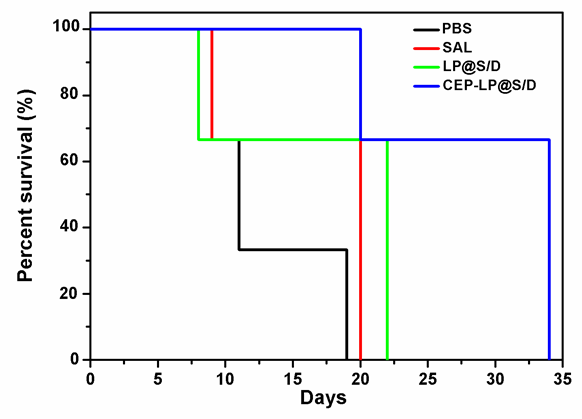
**Supplementary Figure 8.** H&E staining analysis of heart, liver, spleen, lung, kidney of tumor bearing xenograft mice after treatment. Scale bar =0.1 cm.

**Supplementary Figure 9.** Survival curves for the mice from different treatment groups.
